# Integrating Pavement Sensing Data for Pavement Condition Evaluation

**DOI:** 10.3390/s21093104

**Published:** 2021-04-29

**Authors:** Konstantinos Gkyrtis, Andreas Loizos, Christina Plati

**Affiliations:** Laboratory of Pavement Engineering, NTUA Campus, National Technical University of Athens (NTUA), 5, Iroon Polytechniou, GR-15773 Athens, Greece; aloizos@central.ntua.gr (A.L.); cplati@central.ntua.gr (C.P.)

**Keywords:** pavement profile, deflectometric data, geophysical inspections, data integration, pavement management

## Abstract

Highway pavements are usually monitored in terms of their surface performance assessment, since the major cause that triggers maintenance is reduced pavement serviceability due to surface distresses, excessive pavement unevenness and/or texture loss. A common way to detect pavement surface condition is by the use of vehicle-mounted laser sensors that can rapidly scan huge roadway networks at traffic speeds without the need for traffic interventions. However, excessive roughness might sometimes indicate structural issues within one or more pavement layers or even issues within the pavement foundation support. The stand-alone use of laser profilers cannot provide the related agencies with information on what leads to roughness issues. Contrariwise, the integration of multiple non-destructive data leads to a more representative assessment of pavement condition and enables a more rational pavement management and decision-making. This research deals with an integration approach that primarily combines pavement sensing profile and deflectometric data and further evaluates indications of increased pavement roughness. In particular, data including Falling Weight Deflectometer (FWD) and Road Surface Profiler (RSP) measurements are used in conjunction with additional geophysical inspection data from Ground Penetrating Radar (GPR). Based on pavement response modelling, a promising potential is shown that could proactively assist the related agencies in the framework of transport infrastructure health monitoring.

## 1. Introduction

Being a core part of the critical transportation infrastructure network, highways serve the need for safe transportation of human beings and freight on a daily basis at both national and international level. As such, modern roadways constitute fundamental core investments and significant assets indicating economic health and social prosperity [1]. To maintain this prestige, roadways require pavements in good overall physical condition with both structural soundness and increased serviceability. The latter is the most important factor that road users can judge, since users’ satisfaction is related to pavement ride comfort and road safety considerations that both affect driving quality [2,3,4,5,6]. This is why highway pavements are most often monitored in terms of their surface performance considering that the major cause that triggers maintenance is reduced pavement serviceability due to surface distresses, excessive pavement unevenness and/or texture loss.

Among the surface condition indicators, pavement roughness is a critical one, which apart from indicating surface comfortability affects vehicles’ movement and speed, fuel efficiency and general vehicle costs [3,7,8,9,10]. In addition, it has been reported that roughness characterizes energy consumption during the use phase from a life-cycle assessment view [11]. These facts justify why roughness measurements attract the interest of a Pavement Management System (PMS) [12,13]. Roughness is most often quantified in terms of the International Roughness Index (IRI, m/km), developed by the Word Bank in the mid-1980s as a standardized measurement method [14]. Vehicle-mounted laser profilers (or Road Surface Profilers, RSPs) constitute popular and practical systems with high productivity that are used to sense pavement profile data. RSPs can move at traffic speeds up to 80 km/h, so big data can be rapidly and cost-effectively sensed without the need for traffic interventions. RSP’s output is a direct input into a PMS that assists the decision-making processes.

Over recent decades, there have been many studies attempting to extend the power of IRI by investigating interdependencies between roughness and structural indices or other pavement surface distresses, such as cracking or rutting [15]. Most focus on the relationship between IRI and Pavement Condition Index (PCI), which is an indicator of surface condition, based on linear regression modelling and machine learning techniques [9,16,17]. The rationale behind this approach lies upon the fact that the relationship between roughness and pavement distresses is bilateral [17,18]. Rough surfaces might tend to increase the pavement vertical stresses, impose surface deformations and exacerbate pavement fatigue [17]. Vice versa, a distressed pavement will progressively deteriorate pavement roughness [19]. Besides, surface profile imperfections are known to modify the load magnitude of moving vehicles that oscillate vertically because of vehicle dynamics [20]. Several pieces of research have so far focused on vehicular dynamic loading [20,21,22], since the interaction of truck suspension system with pavement profile may exert additional forces to the pavement structure [23]. Even at smooth profiles, pavement is subject to traffic loads of around 10% higher than the design loads, and as a consequence pavement damage acceleration might be expected earlier [21]. In the same context, a reduction of about 29% and 20% has been reported for bottom-up fatigue failure and subgrade rutting failure, respectively [20].

However, including pavement-vehicle dynamics into common analysis aspects is peculiar, so research interests are consistently centered on simpler correlations between roughness and pavement structural performance [15,16,18,24]. The rationale of such approaches is grounded on the speed limitations, high unit costs and stop-and-go impacts on traffic when using stationary deflectometric systems, such as the Falling Weight Deflectometer (FWD) or the Heavy Weight Deflectometer (HWD). The main idea is to evaluate structural indices alternatively in order to reduce the testing frequency of slow and expensive systems [24]. However, no consistent results have been reported and it has been proved rather laborious to develop analytical relationships that could be directly usable by the related agencies. Indeed, according to a Federal Highway Administration (FHWA) report based on Long-Term Pavement Performance (LTPP) data [25], no relationship was found between IRI and the Structural Number (SN), neither in the parameter values nor in their change rates. These remarks coincide with the authors’ perception that the stand-alone use of RSPs cannot provide the related agencies with information with respect to a pavement’s structural integrity despite their cost-effectiveness for network level investigations. This is even more pronounced on highways with limited or no surface deterioration, as is the case with Public Private Partnerships (PPP) highways where the related agencies strive to continuously maintain structurally sound and fully serviceable pavements. Destructive testing, such as coring, cuts and boreholes, are rather undesirable, because they are time-consuming, costly and provide location-specific information. On the contrary, NDT and/or sensor-based structural assessment appear to be better approaches, which are more than necessary in order to proactively detect potential issues that could progressively become obvious in the pavement surface in due course.

A reliable determination of pavement structural condition deterioration over time under traffic and environmental loading can be established through sensing pavement deflections with either the FWD or the more sophisticated Traffic Speed Deflection Devices (TSDD) [26,27,28]. As a standard practice worldwide, deflectometric data is most often combined with pavement stratigraphy data estimated through geophysical surveys, i.e., Ground Penetrating Radar (GPR), in order to back-calculate the pavement stiffness profile and further proceed with a pavement response and failure analysis [26,29,30,31]. Thus, the integration of systems utilized for both the structural and functional assessment of pavements is more than desirable, since a good ride quality itself may not necessarily ensure structural adequacy. On the other hand, even in cases of roughness issues with no other surface distresses, the absence of structural information may result in erroneous maintenance planning. An example is the case of pavement resurfacing that can immediately reduce roughness and restore pavement serviceability without significantly improving pavement structural capacity [24]. In addition, the presence of roughness might sometimes indicate pavement construction issues (e.g., improper design or poor compaction) and structural problems that may not necessarily be obvious in the pavement surface [25]. In other words, the integration of both RSP and FWD might lead to a more holistic assessment of pavement assets during their lifespan. Besides, this is the purpose of integrating multiple non-destructive testing systems in order to sense the pavement condition and manage transportation assets both proactively and cost-effectively in terms of maintenance or rehabilitation planning.

## 2. Aim and Objectives

On these grounds, the present research study aims to assess how the presence of roughness issues along an asphalt pavement surface with no other surface distresses could indicate hidden structural issues within one or more pavement layers or even issues within the pavement foundation support. Towards this, LTPP data from a trial pavement section of an interurban motorway was used to develop an integrated analysis approach within the framework of proactive monitoring of pavement assets. The considered sensing data included roughness measurements through RSP, deflectometric data through FWD and pavement thickness data estimated through GPR analysis. Although pavement profile is known to interact with pavement response in terms of pavement–vehicle dynamics, the present investigation focuses on static loading conditions that are usually adopted in the framework of pavement analysis as a standard practice [26,29,30]. Therefore, to meet the research aim, the following objectives were set:

- To statistically treat RSP data in order to define characteristic IRI values at the FWD test locations.
- To integrate FWD and GPR data, model the pavement response and calculate pavement critical strains.
- To investigate pavement strain modelling aspects based on mechanistic principles considering both deflections and IRI values as input.
- To assess the findings of the modelling process by investigating alternative pavement models and to demonstrate the power of integrating sensing data as an effective solution towards reliable decision-making for transport infrastructure health monitoring.

## 3. NDT-Based Pavement Sensing

### 3.1. Roughness—Road Surface Profiler (RSP)

**Figure 1:**
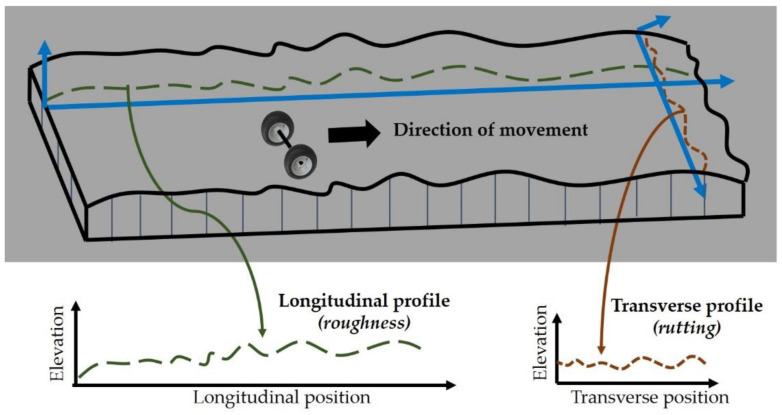
Schematic illustration of pavement profile components (adapted from [33], copyright 2018, Fast track publications).

The presence of surface vertical irregularities and profile elevations along a pavement’s longitudinal line contributes to what is defined as pavement roughness (Figure 1). Roughness is responsible for a vehicle’s suspension response while it moves over the road [32]. IRI calculations are based on the dynamic response of a reference automobile, the so-called Quarter-Car System (QCS) [3,5,32,33]. The model simulates a QCS travelling at a constant speed of 80 km/h and measures the suspension deflection. IRI is calculated as the cumulative vertical movement of the suspension of the QCS divided by the traveling distance, resulting in an index with units of slope, m/km or mm/m [32]. When IRI equals zero, the pavement surface is completely smooth (even), while an IRI value of more than 8 m/km implies that a pavement is practically impassable (uneven), requiring low vehicle speeds [34].

IRI was developed to be workable with different measurement systems or techniques. Nevertheless, the use of high speed inertial RSPs outperforms any other relevant systems or methods (e.g., straight edge, Dipstick profiler, profilograph) [3]. RSPs operate at high speeds, can detect and analyze long wavelengths, exhibit excellent repeatability and their output is a direct measurement of the actual pavement profile. They consist of: (i) a supporting frame, to which laser sensors are attached, suspended approximately 30 cm above the pavement surface, (ii) an odometer and (iii) an inertial unit (accelerometer) that detects vehicle movement in the vertical plane [33]. The latter establishes a moving reference measurement plane and makes the produced data practically independent of the RSP speed, provided that no sharp speed changes occur.

RSP sensor systems have in general a constant performance and the produced profile measurements are reliable [3]. Moreover, since most vehicles travel in well-defined paths, roughness is typically measured at both wheel paths within a traffic lane. Finally, RSPs are also capable of measuring additional surface performance indicators, such as transverse profile, in terms of rut depth (Figure 1) and surface texture.

### 3.2. Load Response—Falling Weight Deflectometer (FWD)

**Figure 2:**
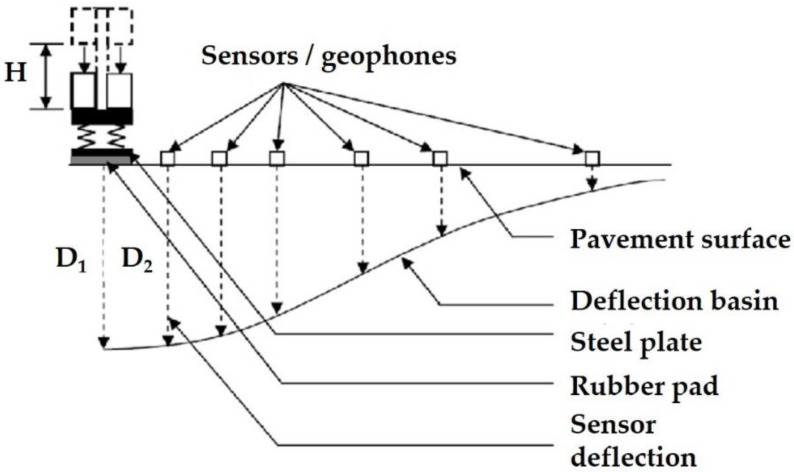
Operation principles of Falling Weight Deflectometer (FWD).

The most popular and practical method for structural integrity assessment of pavement layers and subgrade is the use of the FWD [35,36]. As a stationary device, FWD simulates a moving truck through a load pulse applied to the pavement surface. Dropping a known mass from a specified height (H) onto a steel plate located in the pavement surface results in a typical deflection response, such as that shown in Figure 2. Several load levels can be applied by adjusting the drop height according to the predefined load levels for the LTPP FWD test [37]. The deflection basin at each test location is shaped through surface deflection records, normally achieved through multiple sensors (usually, seven or nine). These sensors are located at specified distances from the center of the loading plate (Figure 2).

A direct consideration of the FWD output leads to deflection-based assessment, which is widely used for a first-level analysis of pavement structural evaluation, useful mainly for network level assessment [31]. A semi-empirical, semi-mechanistic approach was developed for pavement structural analysis, according to which the supplementary use of deflection bowl parameters along with visual inspection surveys facilitate a benchmarking assessment of pavement structure, indicating areas with structural issues [38]. In this context, the first-level analysis is supported by Deflection-Based Parameters (DBPs) that best suit network level investigations. A list of the most commonly utilized DBPs is shown in Table 1 together with the pavement region for which they provide structural indications [38]. In addition, a new methodology has been recently developed for pavement network level assessment based on DBPs without the necessity of knowing layer thicknesses [39].

However, in most cases information about the pavement substructure (i.e., layer thicknesses) is needed in order to proceed with a second-level analysis of pavement condition assessment. In particular, FWD deflections are utilized as input (in combination with layer thicknesses) in order to back-calculate the pavement stiffness profile that is determinant for pavement response analysis [40]. In turn, the pavement failure potential can be assessed, which is necessary in order to estimate pavement remaining life and assess the need for pavement redesign [28,31]. Being a more in-depth analysis, it best suits project level investigations, required, for example, in the framework of potential rehabilitation design strategies. Overall, the FWD system constitutes the standard approach for both project and network level investigations worldwide [26,29,30,36].

### 3.3. Pavement Structure—Ground Penetrating Radar (GPR)

Knowledge of the pavement structure in terms of layer thicknesses provides valuable information for the assessment of both new and in-service pavements. GPR is the most often implemented NDT system for the evaluation of thickness profile based on the dielectric properties of the pavement materials [1,30,41,42,43]. Moreover, the power of GPR as a sensing system, covers additional pavement engineering aspects including, among others, density control during pavement compaction monitoring [44], as well as the assessment of physical properties and pavement subsurface defects [45,46]. Recent reviews of GPR applications in transportation infrastructure can be found in [1,47].

**Figure 3:**
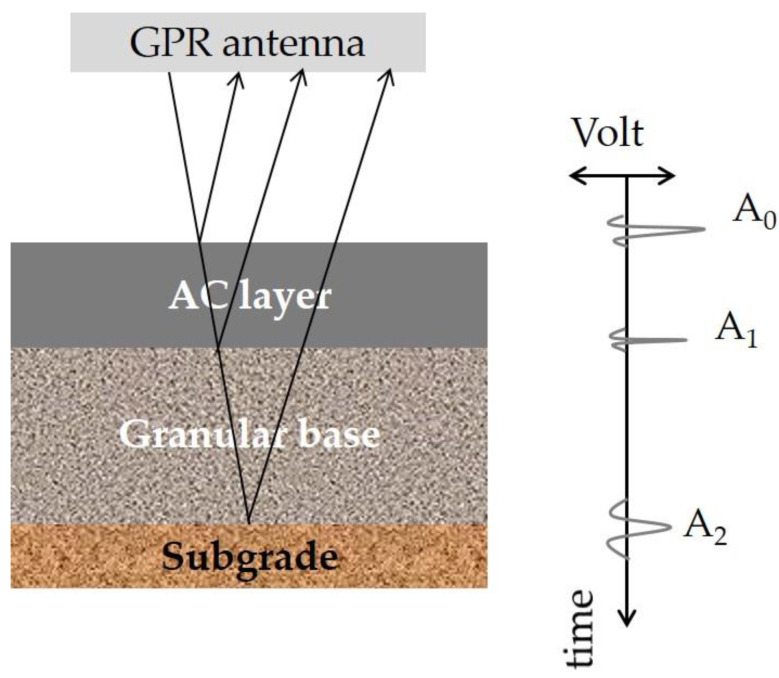
Operation principles of Ground Penetrating Radar (GPR) (adapted from [28]. Copyright 2020 Elsevier).

GPR generates high frequency pulsed electromagnetic waves that penetrate the pavement structure (Figure 3). In particular, a transmitting antenna radiates an electromagnetic wave, whose velocity is affected by the electrical properties of the investigated pavement materials. When the wave reaches a boundary with different electrical properties, a portion of the energy continues to transmit, while another part is reflected backwards and a receiving antenna captures the reflected signal. Typically, 1 GHz and 2 GHz air-coupled antennae are most commonly used [44,48], with a typical penetration depth of approximately 0.5–0.9 m.

According to Figure 3, peak amplitudes A_0_, A_1_ and A_2_ indicate pulse reflections at the pavement layers’ interfaces. Considering, for example, the Asphalt Concrete (AC) layers, the time interval between peaks A_0_ and A_1_ refers to the two-way travel time of the signal within this layer (t_AC_). Further, the AC dielectric constant (ε_AC_) can be estimated based on the surface reflection method [49] as:

(1) $$\varepsilon_{\mathrm{AC}}={\left(\frac{1+\frac{A_{0}}{A_{P}}}{1-\frac{A_{0}}{A_{P}}}\right)}^{2}$$

where A_P_ is the amplitude of an incident GPR signal transmitted onto a flat metallic plate at the pavement surface for calibration purposes. GPR raw data are frequently filtered in order to achieve signal amplification and remove any noise that may affect signal quality and thus the accuracy of dielectric constant estimations [50]. In particular, vertical filtering is applied to deal with local noise and interference removal or random high-frequency noise acting as a bandpass filter in the time domain [28]. Horizontal filtering corresponds to the spatial sampling frequency and it is applied to smooth sharp or rapid changes in the signals. Horizontal scans can be as low as five [28], or as high as 400 to ensure increased computational efficiency [51]. Thereafter, the AC thickness can be determined as follows (c is the speed of light in vacuum):

(2) $$h_{\mathrm{AC}}=\frac{c\cdot t_{\mathrm{AC}}}{2\cdot \sqrt{\varepsilon_{\mathrm{AC}}}}$$

Obviously, inaccuracies in thickness assessment result in erroneous response analysis and life expectancy estimation with detrimental financial impact during maintenance planning [1]. However, there is enough evidence with respect to the increased GPR accuracy of thickness estimations based on comparison with ground-truth data, or coring [28]. In this context, the use of GPR complements pavement condition assessment as a standard evaluation approach [28,30,46].

## 4. Test Site and LTPP Data Collection

**Figure 4:**
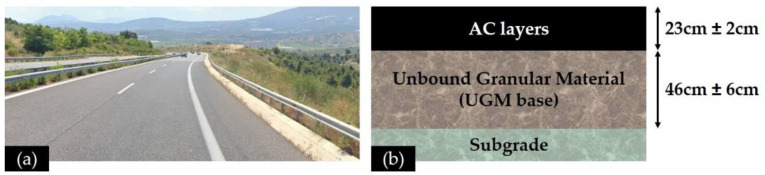
(**a**) Test site, and (**b**) typical pavement cross-section.

LTPP data was collected along the total length of an interurban motorway (Figure 4a). For the purpose of the present research, a 2 km pavement section was selected, located at both cut areas and (mainly) embankments. Roughness issues were easily detectable from road users during the whole pavement’s lifespan. However, since no other indications of surface distresses or deterioration exist, it is believed that the motivation of integrating multiple NDT systems to assess the pavement condition at this area was even further strengthened in order to provide the related highway agencies with a practical and cost-effective monitoring concept.

The pavement structure shown in Figure 4b consists of AC layers, a base of compacted crushed stone materials (Unbound Granular Material—UGM base) and a subgrade layer of natural gravel. With respect to the AC materials, a modified steel slag aggregate mixture and a 4% styrene-butadiene-styrene (SBS) modified binder with a soft bitumen base (80/100 Pen) were used in the wearing course. The achieved penetration grade of the modified binder was 52 Pen and the softening point was 73 °C. A non-modified bitumen with a 50/70 penetration grade and a softening point of 49.5 °C was incorporated into the asphalt base and binder courses. The illustrated thicknesses in Figure 4b refer to the average thicknesses at the 2 km pavement section as estimated from GPR surveys.

**Figure 5:**
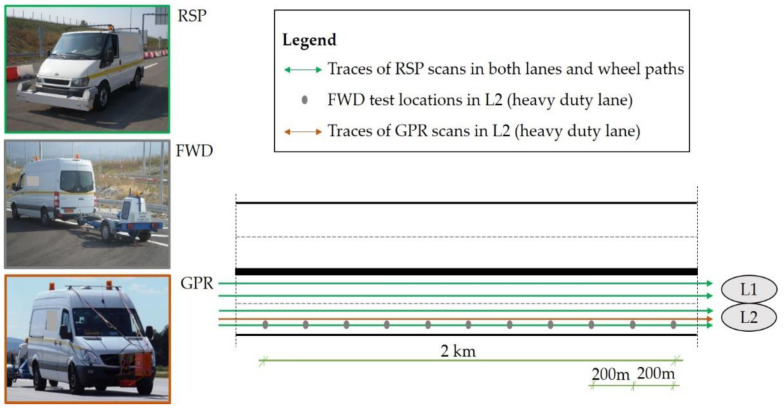
Experimental framework with multiple sensing systems including a Road Surface Profiler (RSP), a Falling Weight Deflectometer (FWD) and a Ground Penetrating Radar (GPR).

An overview of the LTPP experiment is shown in Figure 5. It includes RSP, FWD and GPR measurements according to the monitoring periods shown in Table 2. Year 0 refers to data collection shortly after pavement construction, a process that was repeated once per year for a 7-year monitoring period (years 1–7) in order to assess the pavement performance annually.

GPR measurements (Table 3) along with sample cores were taken at year 0 to define the pavement structure. For the GPR data collection, 10 scans/m were recorded continuously along the right wheel path of the FWD testing along the length of the section (i.e., in the longitudinal direction). Post-analysis of GPR data included AC and base layer thicknesses at 10 m intervals. Compared with ground-truth data (i.e., cores), the GPR prediction error was found to range from 1–8%, which is an acceptable range in accordance with other relevant studies [28,52].

Pavement surface roughness data was sensed in both lanes (L1 and L2) and wheel paths. In particular, the sensor system used in this study was a vehicle-mounted profiler with seven sensors (i.e., lasers, accelerometers, etc.). Roughness was recorded in both wheel paths as well as along the centerline of the vehicle. The sampling frequency rate of the profiler was approximately 16 kHz and the calculated IRI values were reported at 10 m intervals. However, only data from the right wheel path of the right lane was considered in order to coincide with the FWD measurements at the heavy-duty traffic lane (L2).

Finally, in respect to the FWD tests, surface deflections were recorded in the right wheel path of L2 by nine sensors at radial distances of 0, 200, 300, 450, 600, 900, 1200, 1500 and 1800 mm from the center of a loading plate. A load of 50 kN (708 kPa) was applied at several locations at 200 m intervals. In addition, temperature was systematically measured in the middle of the AC base layer through properly drilled holes within the pavement.

## 5. Analysis

### 5.1. Roughness Data Processing

**Figure 6:**
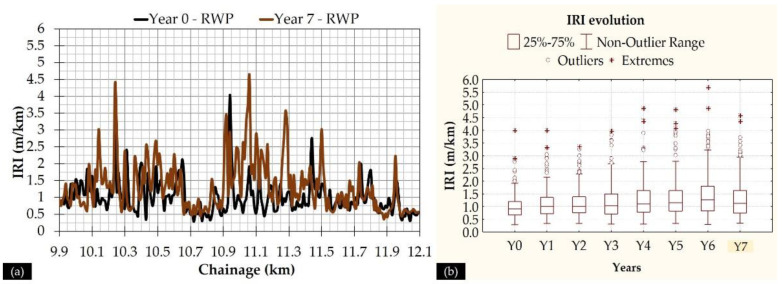
(**a**) Roughness profile along the pavement, and (**b**) roughness evolution yearly in the form of boxplots.

Following the research objectives, the first step was to identify characteristic IRI values for those locations where the FWD testing was applied. An overview of the IRI level along the length of the 2 km section (from chainage +10.0 up to +12.0) is shown in Figure 6. Only the IRI along the Right Wheel Path (RWP) is shown. As can be seen, there is an increased variability across the investigation length and higher IRI values are observed around small and localized areas (Figure 6a). Moreover, there seems to be a tendency for a progressive increase in roughness level (Figure 6b), which is more pronounced for the case of higher IRI values. An exception is observed for Y7, where IRI tends to slightly decrease. This is probably because of a resurfacing that was performed at localized areas before the measurements of Y7. However, as can be seen, IRI is still at high levels in comparison to the first years of pavement’s life. This implies that a simple resurfacing may not fully resolve roughness issues, as has been also mentioned in the literature [24].

**Figure 7:**
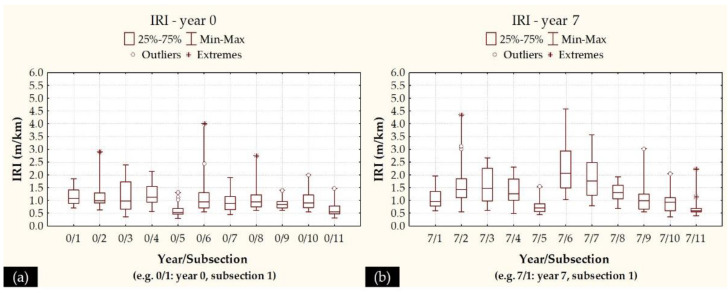
Boxplots of IRI values at the right wheel path (RWP) for (**a**) year 0, and (**b**) year 7.

To further proceed with the integration of roughness and deflectometric data, it was decided to group IRI values of 200 m length (100 m before and 100 m after the exact location of FWD test). Eleven distinct subsections were defined, so that the midpoint of each subsection matches the exact location of the FWD test. Descriptive IRI statistics (only for RWP) are shown in Figure 7 in the form of boxplots for years 0 and 7. The line inside each boxplot refers to the median. Increased variability is again observed, even in the subsections of 200 m, considering the form of boxplots as well as the Coefficients of Variation (CV) at each subsection, shown in Table 4.

As such, an average IRI could not be considered as representative for the whole subsection. In the same context, neither a median IRI itself, nor a characteristic value from a distribution fitting analysis could fully reflect the actual roughness levels at each subsection, due to a lack of information for the higher IRI levels within a subsection. On the other hand, performing denser FWD measurements so that they could match the actual IRI locations was deemed to be laborious and ineffective, since no relevant justification existed for that, due to the absence of other surface deterioration issues (e.g., cracks or rutting) during the pavement’s lifespan. Thus, FWD measurements followed a network level assessment strategy during all monitoring periods and, with respect to roughness, it was decided to select two characteristic IRI values at each subsection to better reflect the pavement profile condition. These included a median IRI value and an “upper” IRI value in order to account for the localized irregularities effect on the near vicinity area. The upper value was defined as:

(3) $$\mathrm{upper}\;\mathrm{IRI}=\max\left\{\begin{array}{c} 90\%\;\mathrm{percentile}\\ \mathrm{IRI}\;\mathrm{at}\;\mathrm{the}\;\mathrm{location}\;\mathrm{of}\;\mathrm{FWD}\;\mathrm{test} \end{array}\right\}$$

**Figure 8:**
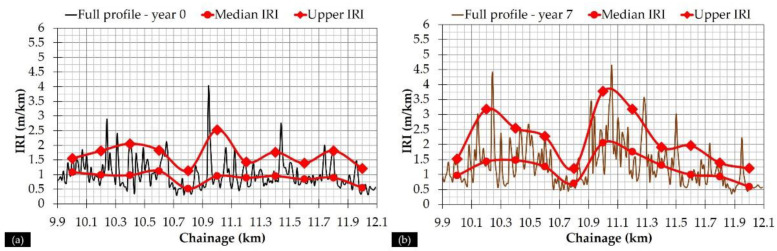
Full pavement profile and characteristic IRI values for (**a**) year 0, and (**b**) year 7.

Following this approach, both median IRI and “upper” IRI were determined at these 11 subsections for all of the monitoring periods. Two examples of the full pavement profiles, together with the curves depicting the two characteristic set of values, are given for years 0 and 7 in Figure 8.

It can be seen that both characteristic values tend to satisfactorily reflect the full pavement profile. Hereinafter, these two characteristics values (median and “upper”) were used in conjunction with the pavement deflectometric data for pavement assessment.

### 5.2. Deflections

**Figure 9:**
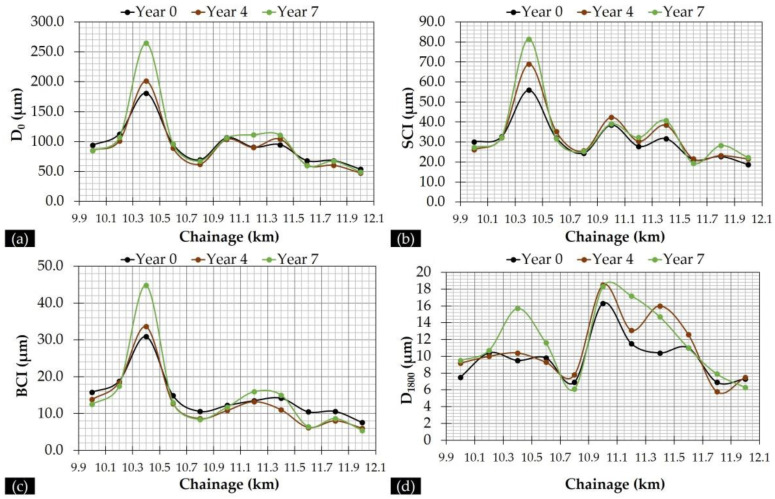
Deflection indexes: (**a**) D_0_, (**b**) Surface Curvature Index (SCI), (**c**) Base Curvature Index (BCI) and (**d**) D_1800_.

Following the indexes presented in Table 1, an overview of the pavement condition is shown in Figure 9 considering four typical indexes and three monitoring periods. From the selected indexes, the D_0_, SCI, BCI and D_1800_ were considered to respectively represent the overall pavement condition, the AC layers condition, the base layer and the subgrade condition.

**Figure 10:**
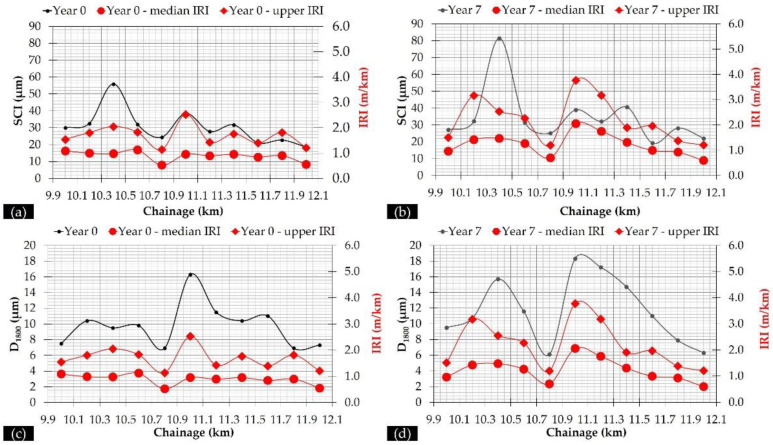
Relationship between (**a**) SCI and IRI, year 0, (**b**) SCI and IRI, year 7, (**c**) D_1800_ and IRI, year 0, and (**d**) D_1800_ and IRI, year 7.

All measured deflections were first normalized at the target load of 50 kN and an additional temperature normalization at the temperature of 20 °C was performed for D_0_ and SCI according to [53]. As such, individual D_0_ and SCI curves became comparable. Given this, a slightly progressive deterioration was observed for the overall pavement condition as well as for the individual layer behaviour. In addition, the evolution trend of D_0_ is similar to that of SCI and BCI indexes. Contrariwise, the evolution of the D_1800_ index seems to be less reflected in the D_0_ index. Further, the relationship between roughness and deflectometric data is presented in Table 5 in terms of the square of the correlation coefficient (R^2^) value and in Figure 10, where the evolution of SCI and D_1800_ indexes is shown in conjunction with roughness.

From the R^2^ values, it seems that a yearly variance exists amongst the observed correlations. In particular, D_0_, SCI and BCI indexes (related to pavement layer condition) exhibited a better correlation with IRI_upper_ than IRI_median_, whereas the D_1800_ (related to pavement subgrade condition) exhibited moderate to good correlation with both IRI values. Moreover, from Figure 10c,d is seems that D_1800_ and IRI follow a qualitatively similar evolution trend along the investigation length. Stimulated from these preliminary remarks, a second-level analysis of FWD data was decided upon in order to further investigate the potential IRI contribution to the pavement’s structural performance.

### 5.3. Response Calculations

**Figure 11:**
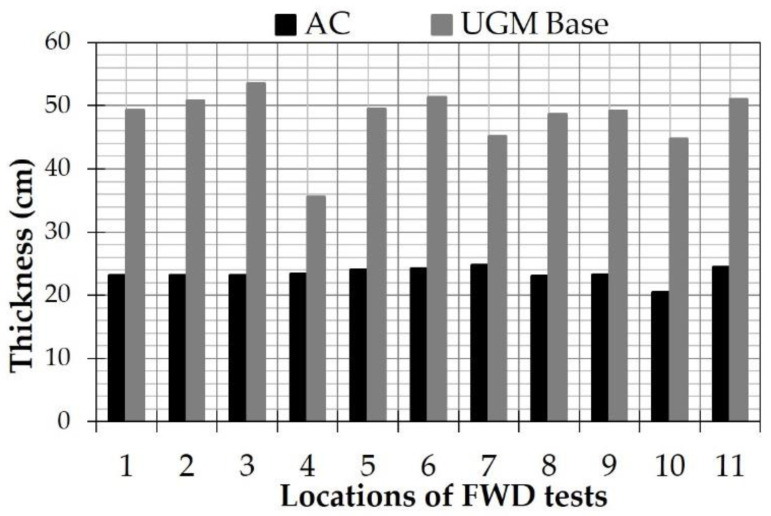
Thicknesses of the Asphalt Concrete (AC) layers and the Unbound Granular Material (UGM) base layer.

For pavement response analysis, two separate processes were followed. At first, deflectometric data was integrated with GPR-based thicknesses (shown in Figure 11) in order to back-calculate the pavement stiffness profile for all monitoring periods according to [54]. A typical three-layered system (Model A, as per Figure 4b) was initially considered following the commonly adopted approach for pavement analysis. Second, the pavement stiffness profile was used to generate pavement response data (i.e., strains) against loading according to [55]. Both procedures were based on the Multi-Layer Elastic Theory (MLET), assuming all material behaviour as linear elastic according to a worldwide-applied assumption [26,30,31,40]. This was further strengthened herein, because of the absence of any kind of deterioration (e.g., cracks) that could adversely affect the moduli reasonability or compatibility with continuum mechanics.

Following MLET core principles, seed moduli values are assigned to the individual pavement materials and a theoretical deflection bowl is calculated. Additional input data related to field AC temperature, layer thicknesses and layer Poisson’s ratio (assumed to be 0.35) are important for the analysis. Material moduli are iteratively adjusted until an acceptable match between the measured and calculated deflection bowls is achieved. This tolerance level is most commonly controlled through the Root Mean Square (RMS) value that represents the error between measured and calculated deflections. According to Table 6, the interquartile range of RMS is 2.3–4.9, which is in general low.

According to the international literature, the tool of [54] has been proved accurate and consistent in terms of moduli estimation based on frequency distribution plots and CV of moduli, considering data from three replicate FWD levels [56]. Moreover, previous relevant experience demonstrated that the use of [54] produced well-correlated moduli with those predicted with another MLET-based tool [57] and calculated critical strains were found to be in close approximation irrespective of the utilized tool [31].

**Figure 12:**
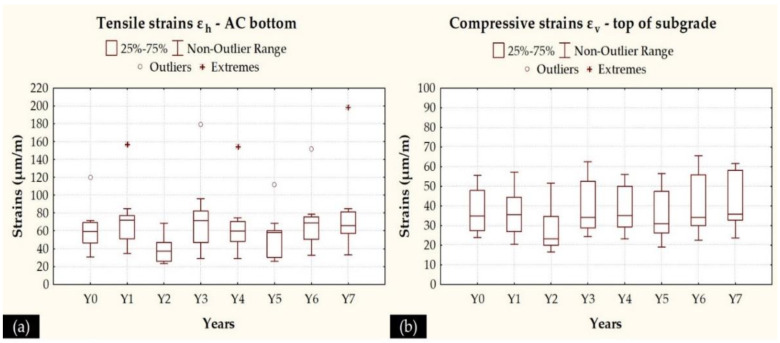
(**a**) Tensile strains at the AC bottom, and (**b**) compressive strains at the top of subgrade.

Strain calculations were performed for both the raw AC temperatures measured in the field as well as at a reference temperature of 20 °C. AC moduli were normalized to the reference temperature according to the algorithm proposed in [54]. For the response calculations, both critical locations were considered, i.e., the bottom of AC layers, related to fatigue failure, and the top of the subgrade, related to permanent deformation failure. A uniform circular loading of 708 kPa was considered during the response calculations and an overview of the resulting horizontal tensile strains (*ε_H_*) and vertical compressive strains (*ε_V_*) at the AC bottom and top of subgrade, respectively, is illustrated in Figure 12 for all monitoring periods.

Pavement condition may be roughly characterized as constant in terms of AC strains (range 40–80 μm/m), apart from the localized deterioration observed through the maximum tensile strains (either an extreme value or an outlier) that coincides with the observed peak values in the deflection indexes curves (Figure 9a–c). With respect to the subgrade condition (strain range 20–60 μm/m), a progressive increase is observed, especially after Y2, which does coincide with the increase in roughness levels observed in Figure 6b. These provided the rationale in order to further investigate potential interaction between deflections and roughness towards strain development through a linear regression analysis.

### 5.4. Pavement Strain Modelling

As a well-known approach, the use of predictive models for the estimation of pavement strains leads to significant time- and cost-savings within pavement analysis, since the time-consuming processes of back-analysis and forward analysis for strain calculations are bypassed [58,59]. Strain modelling enables a rapid screening of pavement structural condition that can in turn enhance maintenance prioritization and decision-making processes [60]. Typically, required inputs for such models include, in most cases, DBPs and, in some cases, layer thicknesses [31]. In this study, the additional incorporation of roughness data was attempted and several linear regression models for critical strain prediction were assessed in terms of both data fit and accuracy evaluation considering the following cases:

- only DBPs used as input (Case I),
- DBPs and median IRI value used as input (Case II),
- DBPs and “upper” IRI value used as input (Case III), and
- DBPs and both characteristic IRI values used as input (Case IV).

Considered DBPs included D_0_, SCI, BDI BCI, D_900_ − D_1200_ and D_1800_. Both strains and DBPs were also considered in logarithmic scale following previous relevant studies [31,60]. AC temperature (T_AC_) was used as an additional input parameter in all cases. Given this, the following generalized relationship was used as reference during the regression analysis (a_1_, … a_9_ are regression constants):

(4) $$\log\varepsilon =a_{1}+a_{2}\log D_{0}+a_{3}\log\mathrm{SCI}+a_{4}\log\mathrm{BDI}+a_{5}\log\mathrm{BCI}+a_{6}\log\left(D_{900}-D_{1200}\right)\;+a_{7}\log D_{1800}+a_{7}\log T_{\mathrm{AC}}+a_{8}{\mathrm{IRI}}_{\mathrm{median}}+a_{9}{\mathrm{IRI}}_{\mathrm{upper}}$$

In terms of the modelling process, it is noted that data from years 0, 1 and 2 were used for model calibration (i.e., 37.5% of the total data) and data from years 3–7 (i.e., the rest 62.5%) were used for model accuracy evaluation. Through this discrimination, temperatures covering the full spectrum measured in-situ (i.e., 14–30 °C) were taken into consideration. The model calibration was evaluated based on the R^2^ value, whereas model accuracy was assessed through the Root-Mean-Square-Percentage-Error (RMSPE %), calculated as follows:

(5) $$\mathrm{RMSPE}\left(\%\right)=\sqrt{\frac{\sum_{i=1}^{n}{\left(\frac{\varepsilon_{\mathrm{pred}}-\varepsilon_{\mathrm{calc}}}{\varepsilon_{\mathrm{calc}}}\right)}^{2}}{n}}\cdot 100$$

where:

- ε_pred_: strains predicted through models (μm/m),
- ε_calc_: strains calculated through MLET (μm/m), and
- n: observations (i.e., number of deflection basins under consideration).

From a stepwise process, only parameters that were statistically significant (*p* values less than 0.05) were considered as strain predictors and the results of the linear regression models for the estimation of strains are shown in Table 7.

**Figure 13:**
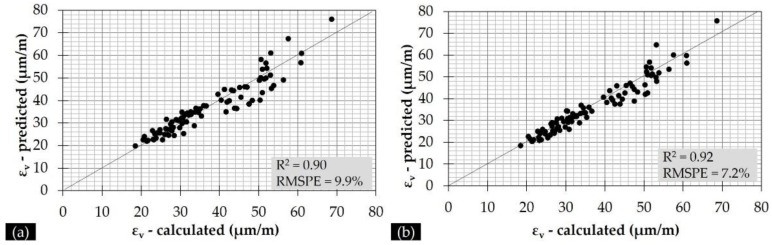
Calculated versus predicted subgrade strains considering as input: (**a**) DBPs (Case I), and (**b**) DBPs and IRI level (Case IV).

It can be seen that both characteristic IRI values do not affect the AC strains, since no different models were found for Cases II–IV. Only a negligible improvement in terms of model accuracy was found in Case II for AC strains at the raw measured temperatures T (°C). On the other hand, pavement roughness was found to have a strong impact on subgrade strains, since in many of the investigated cases a different model was found with both a better fit and a better accuracy. In particular, the use of both characteristic IRI values (Case IV) for subgrade strains at the raw measured temperatures T (°C) led to a model with increased accuracy (Figure 13) compared to that with the sole use of DBPs (Case I).

Further, even the addition of the median IRI itself (Case II) led to an improvement in the subgrade strain prediction accuracy in comparison to Case I. However, the “upper” IRI value itself (Case III) was not found to affect subgrade strain levels. This coincides with the lower correlation observed between the D_1800_ index and the IRI_upper_ compared to the pair of D_1800_-IRI_median_ (as shown in Table 5). Perhaps the IRI_upper_ could affect the subgrade strains in case of performing FWD tests at the exact localized areas where IRI issues are more pronounced.

Overall, the remarks initially made for the correlations between deflection indexes and IRI values were modified during the strain calculations. In particular, AC strains were found rather insensitive to roughness levels, whereas subgrade strains exhibited some kind of dependency on roughness. It became feasible to highlight this interaction between the structural and functional performance of the experimental pavement based on the integration of multi-sensing data that were applied to prove their interrelationship.

## 6. Discussion Points and Assessment of Findings

Pavement condition is usually evaluated by measuring the ride quality or roughness, surface distress, structural adequacy and pavement friction [2,16,30,46,61,62]. Towards this, different NDT systems are utilized to sense the pavement condition. Since the stand-alone use of these systems provides limited information, the integration of multiple systems helps to reach a reliable pavement health monitoring and eventually reliable decision-making regarding pavement condition.

**Figure 14:**
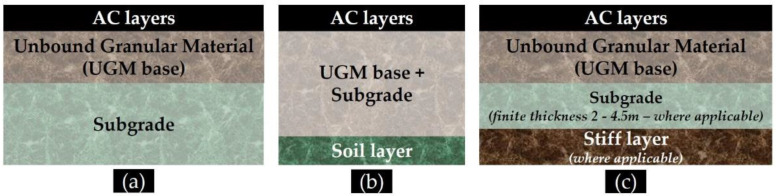
Pavement models for response analysis: (**a**) Model A: AC/UGM/SUBG (reference model), (**b**) Model B: AC/UGM + SUBG/SOIL, and (**c**) Model C: AC/UGM/SUBG/STIFF SOIL.

This study dealt with the potential relationship between roughness issues and pavement structural condition. The focus was on an experimental pavement section, where roughness issues existed along its length even during the first monitoring period. The integration analysis of multiple LTPP data, collected through RSP, FWD and GPR, demonstrated that roughness issues might coincide with pavement subgrade condition, since IRI levels were found to be predictors of critical subgrade strains. As such, the present study seems to increase the benefit, supplementary to other research that mainly focuses on the dynamic impact of roughness on vehicular loading response [20,21,22,23], as IRI seems to affect pavement response even during the consideration of static loading conditions that are usually adopted in pavement analysis. Related data is in limited availability within the international literature. Therefore, the present study contributes towards using a practical framework, according to which pavement response and pavement profile are coupled, thereby providing the relevant authorities with an integrated screening approach for areas deserving maintenance focus. To further assess the power of this finding, response analysis was repeated considering two additional pavement models (as shown in Figure 14).

In particular, Model B (Figure 14b) was adopted in order to obtain information for even higher depths. A uniform subgrade layer of 50 cm thickness was assumed along the length of the experimental pavement, and a unified intermediate layer was considered including both UGM base and subgrade which was assumed to lay above a natural soil layer. Thus, the second critical location was now even deeper. In respect to the calculated critical strains, AC strains were again found insensitive to pavement profile variations, whereas vertical strains at the top of the third layer in Model B exhibited the same kind of dependency on pavement profile (similar to Model A). In this case, AC strains were calculated with a 5.9% deviation from those calculated through Model A, whereas vertical strains in the higher depth were reasonably calculated at 40–45% lower than the subgrade strains in Model A.

The third model (Model C, Figure 14c) was similar to Model A, but the effect of a stiff bottom layer was also accounted for. In general, this effect might be neglected when the depth to a stiff layer is greater than 10–12 m [63]. However, this depth is in general unknown and might be verified, whenever possible, with either NDT or borings [63]. Analyzing the deflection bowl itself through a MLET-based tool [57], an automatically estimated depth to a stiff bottom was found for the first three locations, which seems reasonable since these locations were along a cut area. For these locations, back-analysis and response calculations were performed for a four-layer pavement model (shown in Figure 14c). Both critical strains (at AC bottom and top of subgrade) were found to be different from those calculated through Model A, so the impact of a stiff bottom layer on pavement analysis was shown.

Nevertheless, even in this case, pavement profile was again found to be a statistically significant predictor of subgrade strains. In particular, the equation fit and the prediction accuracy for the estimation of vertical strains is shown in Table 8, comparing the results from all pavement models and investigation cases. Further, an overview of the statistical significance of all the considered input parameters (as per Equation (4)) is given in Table 9 for both critical strains. For those variables with *p* value less than 0.05, statistically significant predictors are indicated. From Table 9, the AC temperature was excluded, since the comparison between different pavement response models (A, B and C) was made for strains calculated at raw temperatures.

The values of the regression constants a_1_, … a_9_ (Equation (4)) are not intentionally given, since proper recalibration is needed before using any equation elsewhere. Besides, the purpose of this study was not to generate critical strain predictive equations. It was rather to demonstrate an approach on how to correlate sensing data and obtain integrated information in order to better assess pavement condition and alert agencies for potential intervention actions. This also justifies the author’s choice at this stage for common analytical tools (statistics and regression analysis) instead of a more advanced neural network modelling. To this end, the existence of a predictive equation, even with localized power, based on pavement historical performance and mechanistic-based analysis could be useful.

Overall, pavement roughness is a serious issue that could be in the worst cases related to land subsidence or stability issues. The condition of a pavement-soil system is usually a matter of concern when managing both primary and secondary roadway networks (either paved or unpaved) within a nation’s territory. In such cases, the use of the Synthetic Aperture Radar (SAR) technology could complement the overall process in terms of subsidence monitoring and provide even more cost-effective solutions considering limited fund allocation within the transportation agencies before scheduling other kind of destructive and costly testing. SAR technology has become very popular during the last two decades and numerous applications exist in the domain of infrastructure engineering and monitoring [7,64,65,66].

Nevertheless, even in this case pavement structural performance data should not be absent (if possible), especially in the case of PPP highways. The use of the stationary FWD might be counterbalanced by the use of innovative TSDD [27] for rapid health monitoring. It is believed that the presented methodology could also be applicable for the possible integration of RSP and TSDD, since TSDD will dominate in the near future.

## 7. Conclusions

This research study demonstrated a modelling approach according to which multi-sensing pavement data (i.e., pavement profile, stratigraphy and deflectometric data) was integrated to illustrate a promising monitoring framework and identify additional issues that might be hidden but may often occur at areas with pavement profile issues. Analyzing LTPP data from an experimental pavement with roughness issues along its surface demonstrated that pavement roughness is a significant predictor of critical subgrade strains. In particular, following a modelling approach it was found that the prediction accuracy of subgrade strains was improved when using roughness level as an input additional to DBPs, since a decrease from around 10% to 7–7.5% was observed for the RMSPE index. Contrariwise, the impact on AC strains was found negligible. Three different pavement models that were adopted during the pavement analysis further strengthen the previous remarks. In other words, there is a quantifiable evidence that structural variation within the pavement subgrade (or even deeper) can be somehow reflected in the pavement ride quality. Wherever used as an input parameter for subgrade strain prediction, IRI proved to be a significant indicator with p values less than 0.05.

Since roughness is an important pavement performance indicator, special care should be taken along areas with increased roughness levels by additionally sensing pavement structural performance and better defining the pavement maintenance or rehabilitation strategy. A smart combination of NDT systems and data integration could result in the need for planning denser measurements and help in identifying areas with structural variations, thereby limiting the potential locations that could be subject to other kinds of destructive testing (e.g., cuts or boreholes).

Overall, the presented approach is an initial cost-effective method serving the purpose of transportation assets’ health monitoring. Future research is needed considering greater roadway sections, or sections with additional surface distress issues, such as cracking, in order to investigate how the presented integration approach could behave under different circumstances. In addition, the inclusion of more advanced analysis techniques should be considered for future research (e.g., neural network and machine learning techniques) provided that higher lengths are investigated. Finally, the well-known Power Spectral Density (PSD) method might also be incorporated into the analysis, in order to identify where roughness issues originate in an alternative and more sophisticated way.

## Figures and Tables

**Table 1:** Deflection-Based Parameters (DBPs).

Indexes	Equation	Pavement Region
Central (maximum) deflection: d_0_ [μm]	-	Overall pavement condition
Surface Curvature Index (SCI) [μm]	d_0_ − d_300_	Surface layer condition
Base Damage Index (BDI) [μm]	d_300_ − d_600_	Intermediate layers condition
Base Curvature Index (BCI) [μm]	d_600_ − d_900_	Intermediate layers condition
AREA parameter (AREA) [dimensionless]	6(d_0_ + 2d_300_ + 2d_600_ + d_900_)/d_0_	Overall pavement condition
Area Under Pavement Profile (AUPP) [μm]	0.5(5d_0_ − 2d_300_ − 2d_600_ − d_900_)	Upper layers pavement condition
Deflection at the outer geophone: d_1800_ [μm]	-	Subgrade condition

**Table 2:** Monitoring periods.

NDT System	Years after Construction								Monitoring Periods
	0	1	2	3	4	5	6	7	
RSP	X	X	X	X	X	X	X	X	8
FWD	X	X	X	X	X	X	X	X	8
GPR	X								1

**Table 3:** Ground Penetrating Radar (GPR) test conditions.

Test Condition	Description
Number or GPR scans (scans/m)	10
Length of each scan (km in one file)	10
Road positioning	Right wheel path
Direction of scanning	Longitudinal

**Table 4:** Coefficients of Variation (%) for the IRI at the right wheel path (RWP).

Subsection	Reference Location (FWD Test)	Year 0	Year 7
1	P1 (+10.0)	27%	33%
2	P2 (+10.2)	43%	55%
3	P3 (+10.4)	53%	44%
4	P4 (+10.6)	36%	37%
5	P5 (+10.8)	46%	34%
6	P6 (+11.0)	69%	44%
7	P7 (+11.2)	38%	44%
8	P8 (+11.4)	47%	27%
9	P9 (+11.6)	26%	53%
10	P10 (+11.8)	40%	43%
11	P11 (+12.0)	45%	55%

**Table 5:** R^2^ value between Deflection-Based Parameters (DBPs) and IRI levels.

Pairs	Year 0	Year 1	Year 2	Year 3	Year 4	Year 5	Year 6	Year 7
D_0_-IRI_median_	0.28	0.21	0.51	0.24	0.10	0.23	0.16	0.24
SCI-IRI_median_	0.26	0.19	0.47	0.18	0.10	0.16	0.10	0.21
BCI-IRI_median_	0.25	0.13	0.42	0.13	0.04	0.20	0.08	0.17
D_1800_-IRI_median_	0.12	0.77	0.38	0.30	0.35	0.30	0.52	0.88
D_0_-IRI_upper_	0.37	0.76	0.87	0.67	0.52	0.60	0.76	0.17
SCI-IRI_upper_	0.47	0.71	0.80	0.69	0.52	0.71	0.72	0.12
BCI-IRI_upper_	0.19	0.69	0.88	0.48	0.35	0.54	0.66	0.12
D_1800_-IRI_upper_	0.46	0.49	0.18	0.47	0.33	0.15	0.28	0.69

**Table 6:** Overview of back-analysis results.

Statistics	E_AC_ at 25 °C (MPa)	E_BASE_ (MPa)	E_SUBG_ (MPa)	RMS (%)
Min	1327	83	373	0.6
25%	3095	400	625	2.3
Median	4621	639	778	3.4
75%	5924	908	1021	4.9
Max	16493	2015	1457	12.9
Mean	5180	729	830	3.7
Stand. Dev.	2863	469	281	2.1
CV %	55%	64%	34%	56%

**Table 7:** Regression analysis results.

Strains	Case I	Case II	Case III	Case IV
R^2^ for ε_H_ (AC) at T (°C)	1.00	1.00	Same as Case I	Same as Case II
RMSPE % for ε_H_ (AC) at T (°C)	2.4	2.3	Same as Case I	Same as Case II
R^2^ for ε_H_ (AC) at 20 °C	0.97	Same as Case I	Same as Case I	Same as Case I
RMSPE % for ε_H_ (AC) at 20 °C	0.9	Same as Case I	Same as Case I	Same as Case I
R^2^ for ε_V_ (SUBG) at T (°C)	0.90	0.91	Same as Case I	0.92
RMSPE % for ε_V_ (SUBG) at T (°C)	9.9	8.2	Same as Case I	7.2
R^2^ for ε_V_ (SUBG) at 20 °C	0.75	0.76	Same as Case I	Same as Case II
RMSPE % for ε_V_ (SUBG) at 20 °C	18.0	13.8	Same as Case I	Same as Case II

**Table 8:** Comparison of regression analysis results for three pavement models.

Model (Layers)	Strains	Case I	Case II	Case III	Case IV
A (AC/UGM/SUBG)	R^2^ for $\varepsilon_{V}$	0.90	0.91	Same as Case I	0.92
	RMSPE % for $\varepsilon_{V}$	9.9	8.2	Same as Case I	7.2
B (AC/UGM + SUBG/SOIL)	R^2^ for $\varepsilon_{V}$	0.91	0.92	Same as Case I	0.93
	RMSPE % for $\varepsilon_{V}$	9.8	7.8	Same as Case I	7.4
C (AC/UGM/SUBG or SUBG + STIFF SOIL)	R^2^ for $\varepsilon_{V}$	0.90	0.91	Same as Case I	0.92
	RMSPE % for $\varepsilon_{V}$	11.4	10.8	Same as Case I	9.4

**Table 9:** Synopsis of stepwise regression analysis for all pavement models.

Variable	Model A AC/UGM/SUBG				Model B AC/UGM + SUBG/SOIL				Model C AC/UGM/SUBG or SUBG + STIFF SOIL			
	$\log\varepsilon_{H}$		$\log\varepsilon_{V}$		$\log\varepsilon_{H}$		$\log\varepsilon_{V}$		$\log\varepsilon_{H}$		$\log\varepsilon_{V}$	
	*t* -Value	Sig.	*t*-Value	Sig.	*t*-Value	Sig.	*t*-Value	Sig.	*t*-Value	Sig.	*t*-Value	Sig.
Constant	15.087	0.000	7.778	0.000	10.141	0.000	5.874	0.000	14.712	0.000	1.775	0.080
logD_0_	−11.301	0.000	−6.183	0.000	−3.821	0.000	0.710	0.480	−11.248	0.000	4.535	0.000
logSCI	19.039	0.000	7.203	0.000	18.277	0.000	6.859	0.000	17.632	0.000	2.053	0.043
logBDI	23.488	0.000	8.672	0.000	14.061	0.000	1.707	0.092	21.817	0.000	1.046	0.299
logBCI	12.064	0.000	2.954	0.004	5.297	0.000	−3.160	0.002	10.779	0.000	0.176	0.861
log(D_900_–D_1200_)	5.675	0.000	1.994	0.050	2.185	0.032	0.261	0.795	5.158	0.000	−0.075	0.941
logD_1800_	5.893	0.000	12.090	0.000	1.696	0.094	18.918	0.000	6.783	0.000	8.745	0.000
IRI_median_	2.110	0.038	6.583	0.000	1.059	0.293	7.282	0.000	2.596	0.011	5.342	0.000
IRI_upper_	−1.278	0.205	−2.983	0.004	0.575	0.567	−3.254	0.002	−1.632	0.107	−3.052	0.003

## Data Availability

Not applicable.
